# Author Correction: The evolution and international spread of extensively drug resistant *Shigella sonnei*

**DOI:** 10.1038/s41467-023-38041-3

**Published:** 2023-04-21

**Authors:** Lewis C. E. Mason, David R. Greig, Lauren A. Cowley, Sally R. Partridge, Elena Martinez, Grace A. Blackwell, Charlotte E. Chong, P. Malaka De Silva, Rebecca J. Bengtsson, Jenny L. Draper, Andrew N. Ginn, Indy Sandaradura, Eby M. Sim, Jonathan R. Iredell, Vitali Sintchenko, Danielle J. Ingle, Benjamin P. Howden, Sophie Lefèvre, Elisabeth Njamkepo, François-Xavier Weill, Pieter-Jan Ceyssens, Claire Jenkins, Kate S. Baker

**Affiliations:** 1 NIHR HPRU in Gastrointestinal Infections at University of Liverpool, Liverpool, UK; 2 Department of Clinical Infection, Microbiology, and Immunology; Institute for Infection, Veterinary and Ecological Sciences, Liverpool, UK; 3 Gastro and Food Safety (One Health) Division, UK Health Security Agency, London, UK; 4 Milner Centre for Evolution, University of Bath, Bath, UK; 5 Centre for Infectious Diseases and Microbiology, The Westmead Institute for Medical Research, Westmead, NSW Australia; 6 Western Sydney Local Health District, Westmead, NSW Australia; 7 Faculty of Medicine and Health, University of Sydney, Sydney, NSW Australia; 8 Sydney Infectious Diseases Institute, University of Sydney, Sydney, NSW Australia; 9 New South Wales Health Pathology, Dee Why, NSW Australia; 10 Douglass Hanly Moir Pathology, Macquarie Park, NSW Australia; 11 Centre for Infectious Diseases and Microbiology – Public Health, Institute for Clinical Pathology and Microbiology Research, Westmead Hospital, Westmead, NSW Australia; 12 Department of Microbiology and Immunology, The University of Melbourne at The Peter Doherty Institute for Infection and Immunity, Melbourne, Australia; 13 Institut Pasteur, Université Paris Cité, Unité des Bactéries pathogènes entériques, Centre National de Référence des Escherichia coli, Shigella et Salmonella, Paris, F-75015 France; 14 Division of Human Bacterial Diseases, Sciensano, Ixelles, Belgium

**Keywords:** Infectious-disease epidemiology, Clinical microbiology, Phylogenomics, Epidemiology, Bacterial infection

Correction to: *Nature Communications* 10.1038/s41467-023-37672-w, published online 08 April 2023

In this article the author name Elisabeth Njamkepo was incorrectly written as Elisabeth Njampeko. The original article has been corrected.

