# Supplementary material for: The evolution and international spread of extensively drug resistant Shigella sonnei
Source: Nat Commun. 2023 Apr 8;14:1983. doi: 10.1038/s41467-023-37672-w (PMC10082799; doi:10.1038/s41467-023-37672-w)
Supplement: Supplementary file 1 — Supplementary Information [file 41467_2023_37672_MOESM1_ESM.pdf]

Supplementary Information for: The evolution and international spread of extensively drug resistant *Shigella sonnei*

Supplementary Table 1. Antimicrobial susceptibility and genotypic AMR profiles of a subset of 14 representative isolates. Interpretation was according to European Committee on Antimicrobial Susceptibility Testing (EUCAST) standards.[1] All isolates in this table carry the *dfrA1* and *dfrA17* genes, and have *gyrA*_D87G, *gyrA*_S83L and *parC*_S80I QRDR mutations. Antimicrobials used include ciprofloxacin (CIP), gentamicin (GEN), ceftriaxone (CRO), azithromycin (AZM), mecillinam (MEC), ertapenem (ERT) and trimethoprim-sulfamethoxazole (SXT).

	Minimum Inhibitory Concentrations (MICs) [mg/L]							Genes					
**Accession**	**CIP**	**GEN^**	**CRO**	**AZM**	**MEC**	**ERT**	**SXT**	***bla*$_{CTX-M-27}$**	***erm*(B)**	***mph*(A)**	***qnrB19***	***sul1***	***sul2***
SRR17120129	6	0.75	>32	>256	0.125	0.006	>32	P	P	P	P	P	A
SRR17099017	6	0.75	12	>256	0.125	0.008	>32	P	P	P	P	P	P
SRR17038739	6	0.75	24	>256	0.094	0.006	>32	P	P	P	P	P	P
SRR16940985	6	0.75	16	>256	0.094	0.006	>32	P	P	P	P	P	P
SRR16940973	4	0.75	32	>256	0.125	0.008	>32	P	P	P	P	P	P
SRR16842826	4	0.5	32	>256	0.125	0.006	>32	P	P	P	P	P	A
SRR16842843	6	0.75	32	>256	0.094	0.006	>32	P	P	P	P	P	P
SRR16612616	6	0.75	32	>256	0.094	0.008	>32	P	P	P	P	P	A
SRR15512411	4	0.75	32	>256	0.125	0.008	>32	P	A	P	P	P	A
SRR16598676	6	0.75	0.012	>256	0.094	0.004	>32	A	A	P	P	P	A
SRR16821273	6	0.75	0.012	>256	0.064	0.006	>32	A	A	P	A	P	A
SRR14407961	6	0.75	0.016	12	0.064	0.004	>32	A	A	A	P	A	P
SRR14792720	4	0.75	0.016	>256	0.38*****	0.006	>32	A	P	P	A	P	P
SRR14782977	4	0.75	0.016	>256	0.5*****	0.006	>32	A	P	P	A	P	P

***Microcolonies in zone of inhibition**
^All isolates are susceptible *in vitro*, but this may not translate *in vivo.*

**Susceptible**	**Absent**
**Resistant**	**Present**

Supplementary Table 2. Minimum inhibitory concentration (MIC) against *S. sonnei* 1538171 and *Escherichia coli* MG1655 with and without p1538717_3. Ciprofloxacin (CIP), ertapenem (ERT), mecillinam (MEC), azithromycin (AZM), ceftriaxone (CRO), trimethoprim sulfamethoxazole (SXT) and gentamicin (GEN) were used.

		**MIC (µg/ml)**		
**Antibiotic**	Relevant AMR gene on p893816	*S. sonnei* 1538171 (p1538171_3)	*E. coli* MG1655	*E. coli* MG1655 + p1538171_3
CIP	N/A	6	0.006	0.006
ERT	N/A	0.006	0.008	0.012
MEC	N/A	0.125	0.032	0.064
AZM	*erm*(B), *mph*(A)	>256	3	>256
CRO	*bla*$_{CTX-M-27}$	>32	0.023	16
SXT	*dfrA17*, *sul1*	>32	0.032	0.19
GEN	N/A	0.75	0.38	0.38

**Susceptible**
**Resistant**

Supplementary Table 3. All strains and plasmids and their associated characteristics and references used in the laboratory experiments untaken in this study.

**Strain**	**Characteristics**	**Reference**
Escherichia coli MG1655	Chromosomally encoded kanamycin resistance and GFP.	Kind gift from Michael Bottery (University of Manchester) and used in previous work by Malaka De Silva *et al.,* (2022).[2]
Shigella sonnei:<br><br>SRR17120129, SRR17099017, SRR17038739, SRR16940985, SRR16940973, SRR16842826, SRR16842843, SRR16612616, SRR15512411, SRR16598676, SRR16821273, SRR14407961, SRR14792720, SRR14782977.	Human clinical isolates from the UK. All CipR.MSM5 3.6.1.1.2 genotype.	Provided by UK Health Security Agency (UKHSA).
**Plasmids**		
pKSR100	A conjugative plasmid naturally found in *S. flexneri 2a* 18787_5_65 from the UK (NCBI accession: CP090162)	Baker *et al.,* (2018).[3]<br>Malaka De Silva *et al.,* (2022).[2]
pAPR100	A conjugative plasmid naturally found in a *S. flexneri* 2a isolate from the UK (NCBI accession: CP090161)	Baker *et al.,* (2018).[3]<br>Malaka De Silva *et al.,* (2022).[2]
p1538171_3	A p893816-like conjugative plasmid naturally found in *S. sonnei* 1538171 from the UK (NCBI accession: CP104412)	This study.

Supplementary Table 4. Bioinformatics packages and services used in this study. A table containing names, commands, versions, figures, citations and references for all bioinformatics packages and services used in this study.

Package	Command	Version	Used in	Citation	Reference(s)
BCFTOOLS,[4]	*bcftools*	1.9-80-gff3137d	Figure 2	Danecek, P., et al. (2021).	Danecek, P., et al. (2021). Twelve years of SAMtools and BCFtools. Gigascience 10(2).
BLAST Ring Image Generator (BRIG),[5]	*BRIG*	0.95	S Figure 3	Alikhan, N.-F., et al. (2011).	Alikhan, N.-F., et al. (2011). BLAST Ring Image Generator (BRIG): simple prokaryote genome comparisons. BMC Genomics 12(1): 402.
Burrow Wheeler Aligner (BWA),[6]	*BWA*	0.7.17-r118	Figure 2	Li, H. and R. Durbin (2009).	Li, H. and R. Durbin (2009). Fast and accurate short read alignment with Burrows-Wheeler transform. Bioinformatics 25(14): 1754-1760.
Clinker,[7]	*Clinker*	0.0.21	S Figure 2	Gilchrist and Chooi, (2021)	Gilchrist, C., & Chooi, Y. H. (2021). Clinker & clustermap.js: Automatic generation of gene cluster comparison figures. *Bioinformatics (Oxford, England)*, btab007. Advance online publication. https://doi.org/10.1093/bioinformatics/btab007
Enterobase cgMLST V1 + Hier CC V1, NINJA MJ GrapeTree,[8,9]	*NINJA MJ*	Enterobase: 1.1.3<br>NINJA MJ: 1.0	Figure 1	Zhou, Z., et al. (2018) and Zhou, Z., et al. (2020).	Zhou, Z., et al. (2018). GrapeTree: visualization of core genomic relationships among 100,000 bacterial pathogens. Genome Res 28(9): 1395-1404.<br>Zhou, Z., et al. (2020). The EnteroBase user's guide, with case studies on *Salmonella* transmissions, *Yersinia pestis* phylogeny, and *Escherichia* core genomic diversity. Genome Res 30(1): 138-152.
Fixstart,[10]	*fixstart*	1.5.5	S Figure 2	Hunt *et al.,* (2015)	Hunt, M., Silva, N. D., Otto, T. D., Parkhill, J., Keane, J. A., & Harris, S. R. (2015). Circlator: automated circularization of genome assemblies using long sequencing reads. *Genome biology*, *16*, 294. https://doi.org/10.1186/s13059-015-0849-0
GNU bash,[11]	*bash*	4.3.30	Figure 2	GNU, P (2007).	GNU, P. (2007). Bash, Free Software Foundation: Unix Shell Program.
Gubbins,[12]	*gubbins*	3.2.1	Figure 2	Croucher, N. J., et al. (2015).	Croucher, N. J. *et al.* Rapid phylogenetic analysis of large samples of recombinant bacterial whole genome sequences using Gubbins. *Nucleic Acids Res* **43**, e15-e15, doi:10.1093/nar/gku1196 (2015).
Guppy,[13]	*guppy_basecaller*	3.4.5+f1fbfb	Figure 2	N/A	N/A
Interactive Tree of Life (iTOL),[14]	*N/A*	5	Figures 1 and 2	Letunic, I. and P. Bork (2021).	Letunic, I. and P. Bork (2021). Interactive Tree Of Life (iTOL) v5: an online tool for phylogenetic tree display and annotation. Nucleic Acids Research 49(W1): W293-W296.
IQTREE,[15]	*iqtree*	2.2.0.3	Figure 2	Nguyen, L. T., et al. (2015).	Nguyen, L. T., et al. (2015). IQ-TREE: a fast and effective stochastic algorithm for estimating maximum-likelihood phylogenies. Mol Biol Evol 32(1): 268-274.
MiniMap2,[16]	*minimap*	2.17-r941	Figure 2	Li, H. (2018).	Li, H. Minimap2: pairwise alignment for nucleotide sequences. Bioinformatics 34, 3094-3100, doi:10.1093/bioinformatics/bty191 (2018).
MULTIQC,[17]	*multiqc*	1.12	Figure 2	Ewels, P., et al. (2016).	Ewels, P., et al. (2016). MultiQC: summarize analysis results for multiple tools and samples in a single report. Bioinformatics 32(19): 3047-3048.
MYKROBE,[18]	*mykrobe*	0.11.0	Figure 2	Hunt, M., et al. (2019).	Hunt, M., et al. (2019). Antibiotic resistance prediction for *Mycobacterium tuberculosis* from genome sequence data with Mykrobe. Wellcome Open Res 4: 191.
NCBI-AMRFINDERPLUS,[19]	*amrfinder*	3.10.24	Figure 2	Feldgarden, M., et al. (2021)	Feldgarden, M., et al. (2021). AMRFinderPlus and the Reference Gene Catalog facilitate examination of the genomic links among antimicrobial resistance, stress response, and virulence. Scientific Reports 11(1): 12728.
Picard,[20]	*MarkDuplicates*	2.27.2	Figure 2	Picard Toolkit (2019).	Picard Toolkit. (2019). Broad Institute, GitHub Repository. https://broadinstitute.github.io/picard/; Broad Institute
Pilon,[21]	*pilon*	1.23	S Figure 3	Walker, B. J. et al (2014).	Walker, B. J. et al (2014). Pilon: An Integrated Tool for Comprehensive Microbial Variant Detection and Genome Assembly Improvement. PLOS ONE 9, e112963, doi:10.1371/journal.pone.0112963
PROKKA,[22]	*prokka*	1.14.6	Figure 2<br>S Figure 2	Seemann, T. (2014).	Seemann, T. (2014). Prokka: rapid prokaryotic genome annotation. Bioinformatics 30(14): 2068-2069.
Qualimap,[23]	*multi-bamqc*	2.2.2		García-Alcalde, F. et al., (2012)	García-Alcalde, F. *et al.* (2012) Qualimap: evaluating next-generation sequencing alignment data. *Bioinformatics* **28**, 2678-2679, doi:10.1093/bioinformatics/bts503.
QUAST,[24]	*quast*	5.0.2	Figure 2	Gurevich, A., et al. (2013).	Gurevich, A., et al. (2013). QUAST: quality assessment tool for genome assemblies. Bioinformatics 29(8): 1072-1075.
R,[25]	*R*	4.1.3	Figure 2	R Core Team (2022).	R Core Team (2022). R: A language and environment for statistical computing. Vienna, Austria., R Foundation for Statistical Computing.
Rhier Bayesian Analysis of Genetic Population Structure (RhierBAPS),[26]	*BAPS*	1.0.1	Figures 1 and 2	Tonkin-Hill et al., (2018) and Cheng, Lu et al., (2013).	Tonkin-Hill et al., (2018). RhierBAPS: An R Implementation of the Population Clustering Algorithm hierBAPS. Wellcome Open Research 3 (July): 93. Cheng, Lu et al., (2013). Hierarchical and Spatially Explicit Clustering of DNA Sequences with BAPS Software. Molecular Biology and Evolution 30 (5): 1224–28.
SAMTOOLS,[4]	*samtools*	1.11-4	Figure 2	Li, H. (2011).	Li H. A statistical framework for SNP calling, mutation discovery, association mapping and population genetical parameter estimation from sequencing data. Bioinformatics (2011) 27(21) 2987-93.
SED,[27]	*sed*	4.2.2	Figure 2	GNU, s (2013).	GNU, s. (2013). GNU sed, Free Software Foundation.
Sequencing Read Archive (SRA) Toolkit,[28]	*fastq-dump*	2.11.0	Figure 2	SRA Toolkit Development Team (2022).	SRA Toolkit Development Team (2022). https://trace.ncbi.nlm.nih.gov/Traces/sra/sra.cgi?view=software.
SONNEITYPING script: parse_mykrobe_predict.py,[29]	*[SCRIPT]*	20210201	Figure 2	Holt, K et al. (2021)	Holt, K, Hawkey, J, and Paranagama, K (2021). https://github.com/katholt/sonneityping
TempEst,[30]	*Heuristic residual mean squared*	1.5.3	S Figures 4a and b	Rambaut et al., (2016).	Rambaut, A., Lam, T. T., Max Carvalho, L. & Pybus, O. G. (2016). Exploring the temporal structure of heterochronous sequences using TempEst (formerly Path-O-Gen). *Virus Evolution* **2**, doi:10.1093/ve/vew007
Trimmomatic,[31]	*Trimmomatic*	0.39	Figure 2	Bolger, A. M., et al. (2014).	Bolger, A. M., et al. (2014). Trimmomatic: a flexible trimmer for Illumina sequence data. Bioinformatics 30(15): 2114-2120.
Unicycler,[32]	*unicycler*	0.5.0	Figure 2	Wick, R. R., et al. (2017).	Wick, R. R., et al. (2017). Unicycler: Resolving bacterial genome assemblies from short and long sequencing reads. PLOS Computational Biology 13(6): e1005595.
Unicycler,[32]	*unicycler*	0.4.8	S Figure 3	Wick, R. R., et al. (2017).	Wick, R. R., et al. (2017). Unicycler: Resolving bacterial genome assemblies from short and long sequencing reads. PLOS Computational Biology 13(6): e1005595.

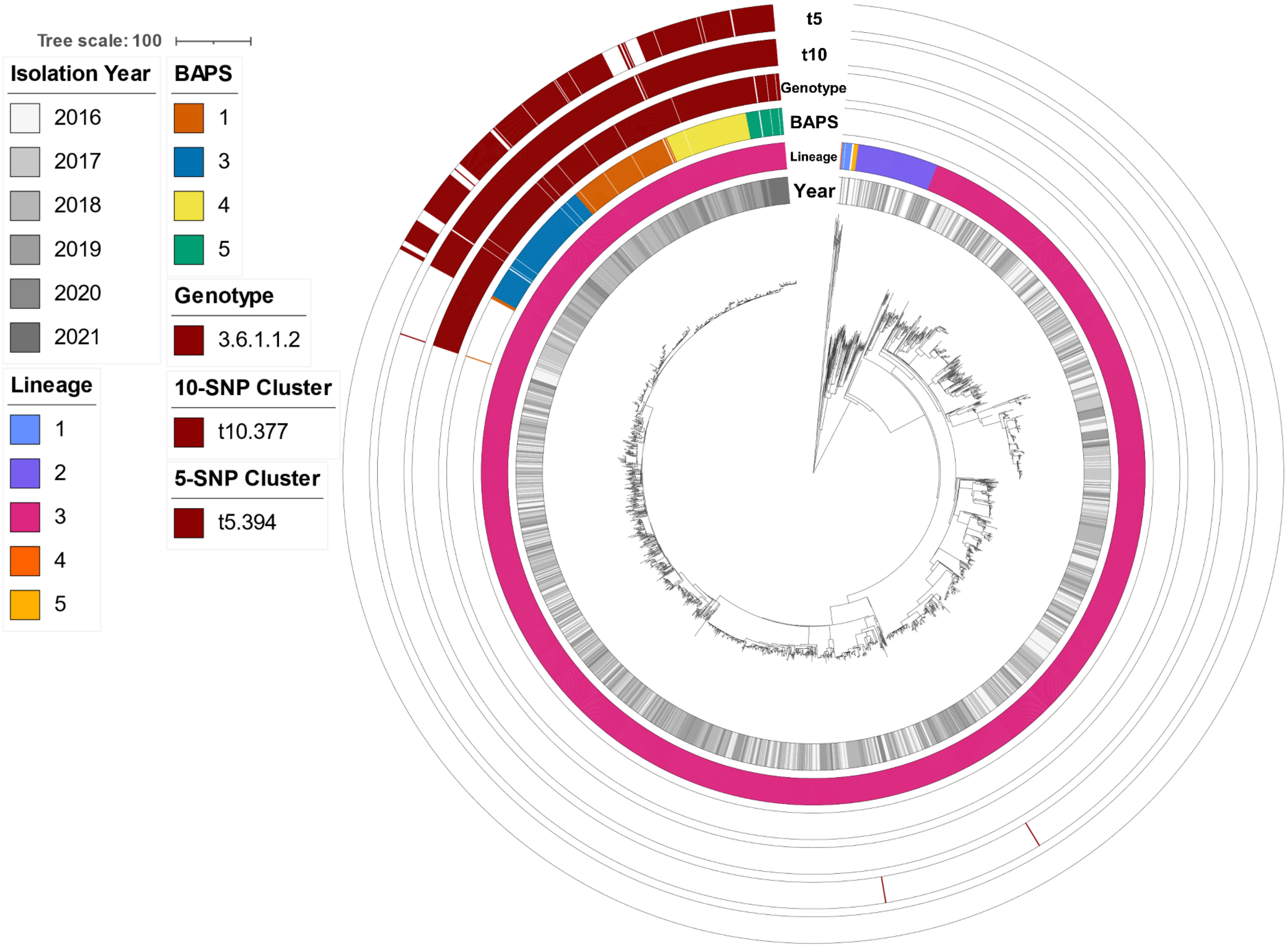

Supplementary Figure 1. Comparison of notation and clustering systems available for *Shigella sonnei*. A cgMLST dendrogram (midpoint rooted) of clinical isolates from the UK (n=2,820) and Lineage subtype references (n=120). Metadata tracks show the year of isolation (for UK isolates only), isolate lineage, BAPS group (for UK t10.377 cluster), and genotype, 10-SNP cluster, and 5-SNP cluster. The scale bar is in cgMLST alleles.

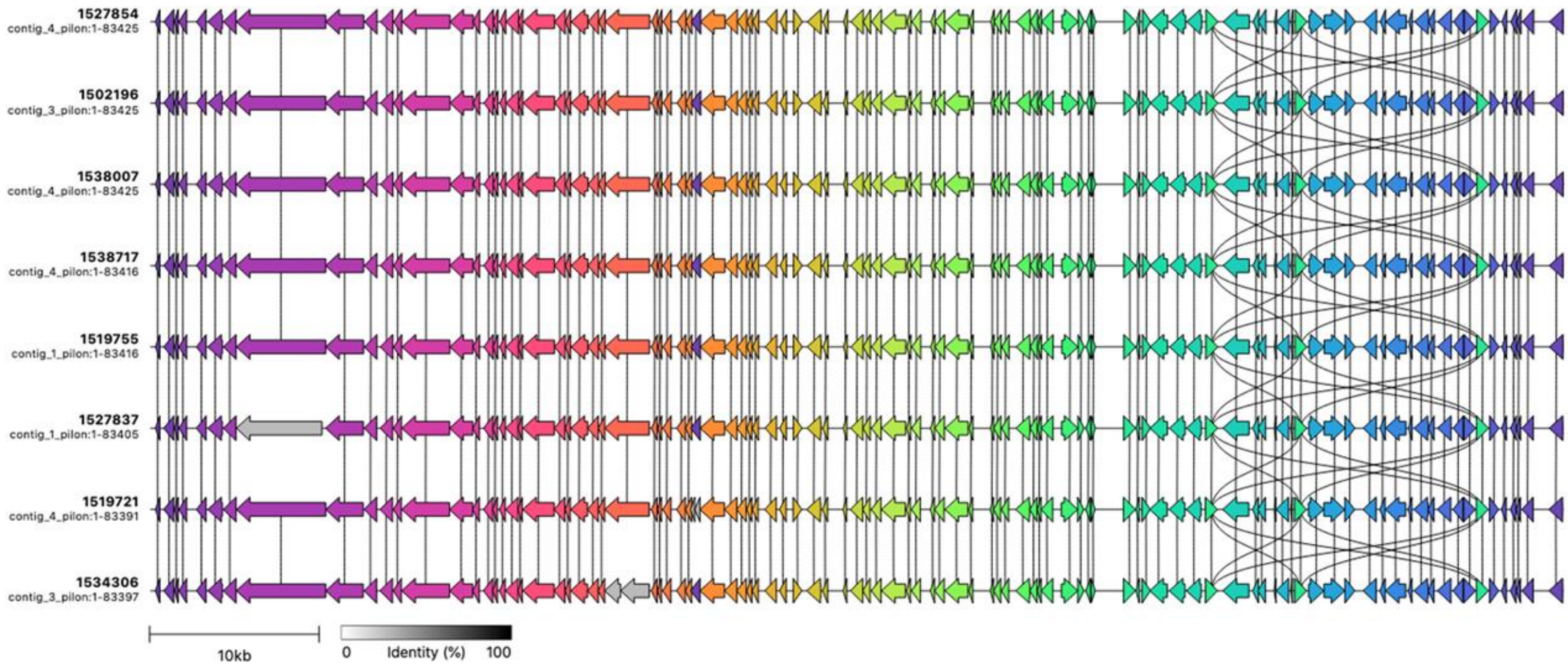

Supplementary Figure 2. Comparison of plasmid p893816 with other sequences Alignment of England, UK, IncFII plasmids harbouring $\textit{bla}_{\text{CTX-M-27}}$. Vertical lines link genes encoding proteins with ≥ 95% sequence similarity. The curved lines on the right-hand side link multiple copies of the IS26 transposase gene present in each plasmid.

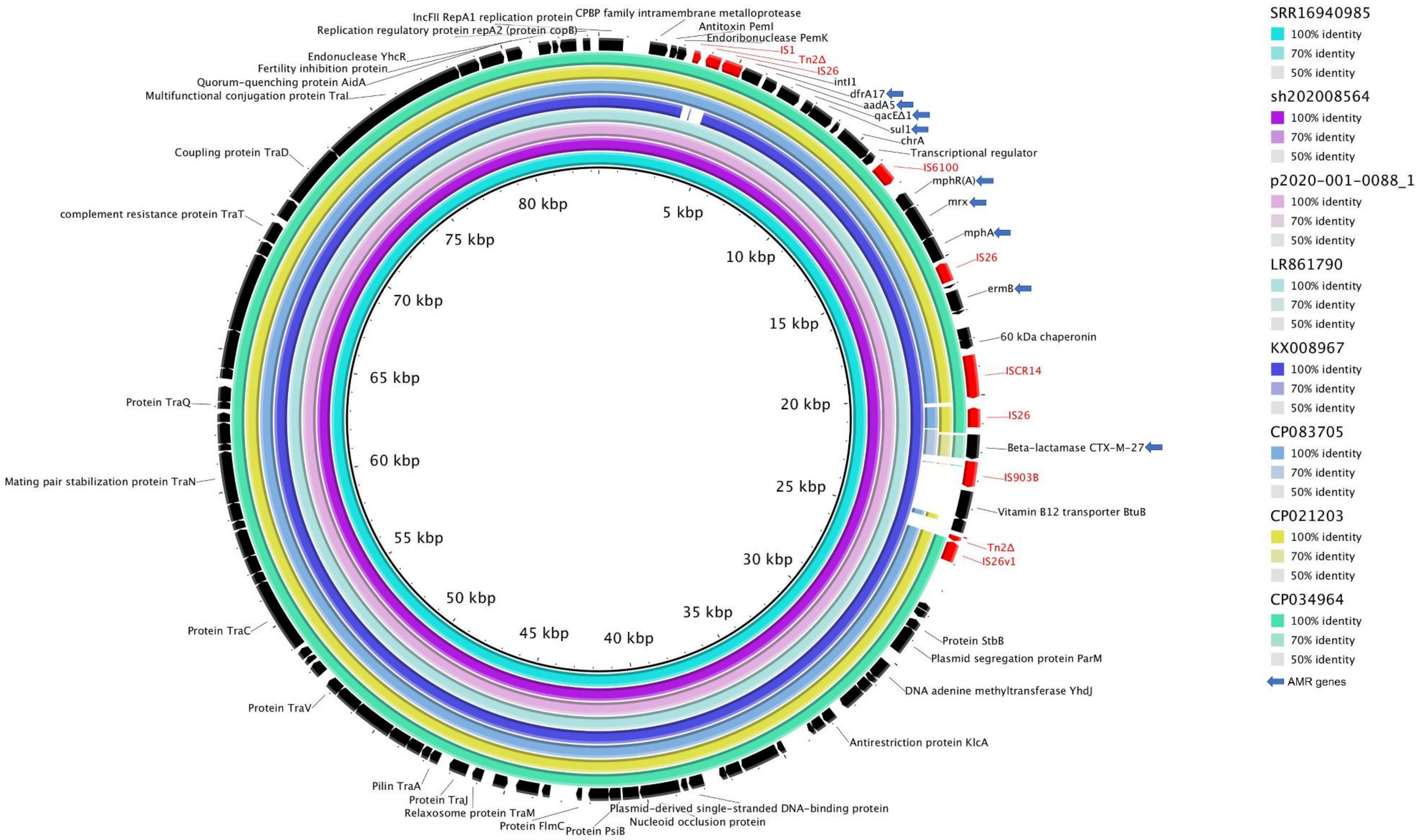

Supplementary Figure 3. Comparison of genes in plasmid p893816 with other plasmids. FII plasmids in BAPS5 isolates carrying *bla*$_{CTX-M-27}$ from different countries are virtually identical to p893816 and other plasmids from the UK and have differences from plasmids in public data. Rings are coloured by plasmid and BLAST similarity according to the inlaid keys, with gene annotations labelled in the outermost ring in black. AMR genes are indicated by blue arrows. IS and transposon fragments are in red. The BRIG plot shows the virtual identity of plasmids in Table 2, differences from p183660 (KX008967) and three other plasmids selected from a BLAST search with > 95% identity, as examples. Note that these three plasmids have large insertions and/or rearrangements vs. p893816 and that segments matching p893816 may not be contiguous in these plasmids. The plasmids shown in the three outermost circles all also carry *dfrA17*, *aad*A5, *mph*(A) and *erm*(B) but have a *bla*$_{CTX-M}$ gene other than *bla*$_{CTX-M-27}$, as indicated by the paler shading (~70% identity; CP083705 and CP021203, *bla*$_{CTX-M-15}$; CP034964, *bla*$_{CTX-M-3}$). These genes all belong to the *bla*$_{CTX-M-1}$ group and have a different origin from *bla*$_{CTX-M-27}$ (*bla*$_{CTX-M-9}$ group).

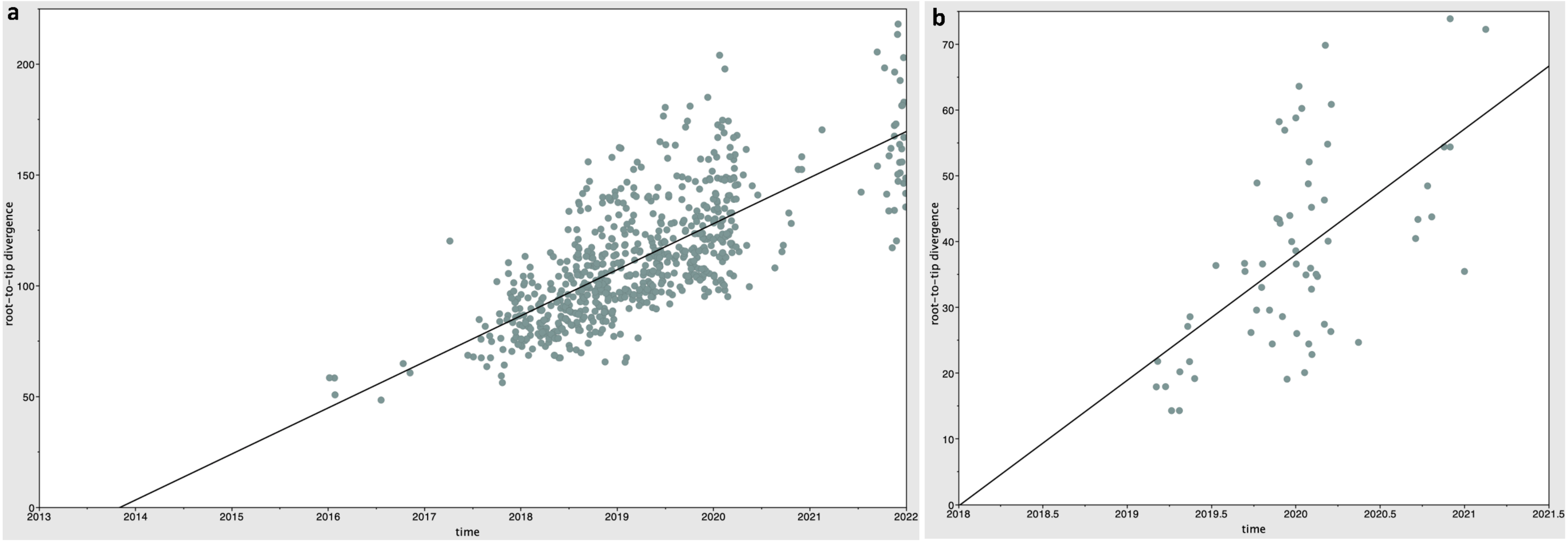

Supplementary Figure 4. Temporal signal analyses of CipR.MSM5 (3.6.1.1.2) isolates. (A) Root-to-tip divergence TempEst,[30] results of all CipR.MSM5 (3.6.1.1.2) isolates from all BAPS groups in England, United Kingdom and New South Wales, Australia. Input was a Newick tree file containing the isolates of interest from Figure 2. The specific day, month and year were known for all isolates. Notably, the incidence of *S. sonnei* declined during the SARS-CoV-2 pandemic. Date range: 5.9809; slope (rate): 20.7715; X-Intercept (TMRCA): 2013.8354, Correlation coefficient: 0.7263; R squared: 0.5274, Residual Mean Squared 366.136. Function: heuristic residual mean squared. **(B)** Root-to-tip divergence TempEst results of CipR.MSM5 (3.6.1.1.2) isolates in the BAPS 5 group from England, United Kingdom and New South Wales, Australia. Input was a Newick tree file containing the isolates of interest from Figure 2. The specific day, month and year were known for all isolates. Date range: 1.9534, slope (rate): 19.0974, X-intercept (TMRCA): 2018.0083, correlation coefficient (0.5946), R squared: 0.3535, residual mean squared 144.905. Function: heuristic residual mean squared. Best-fitting root used.

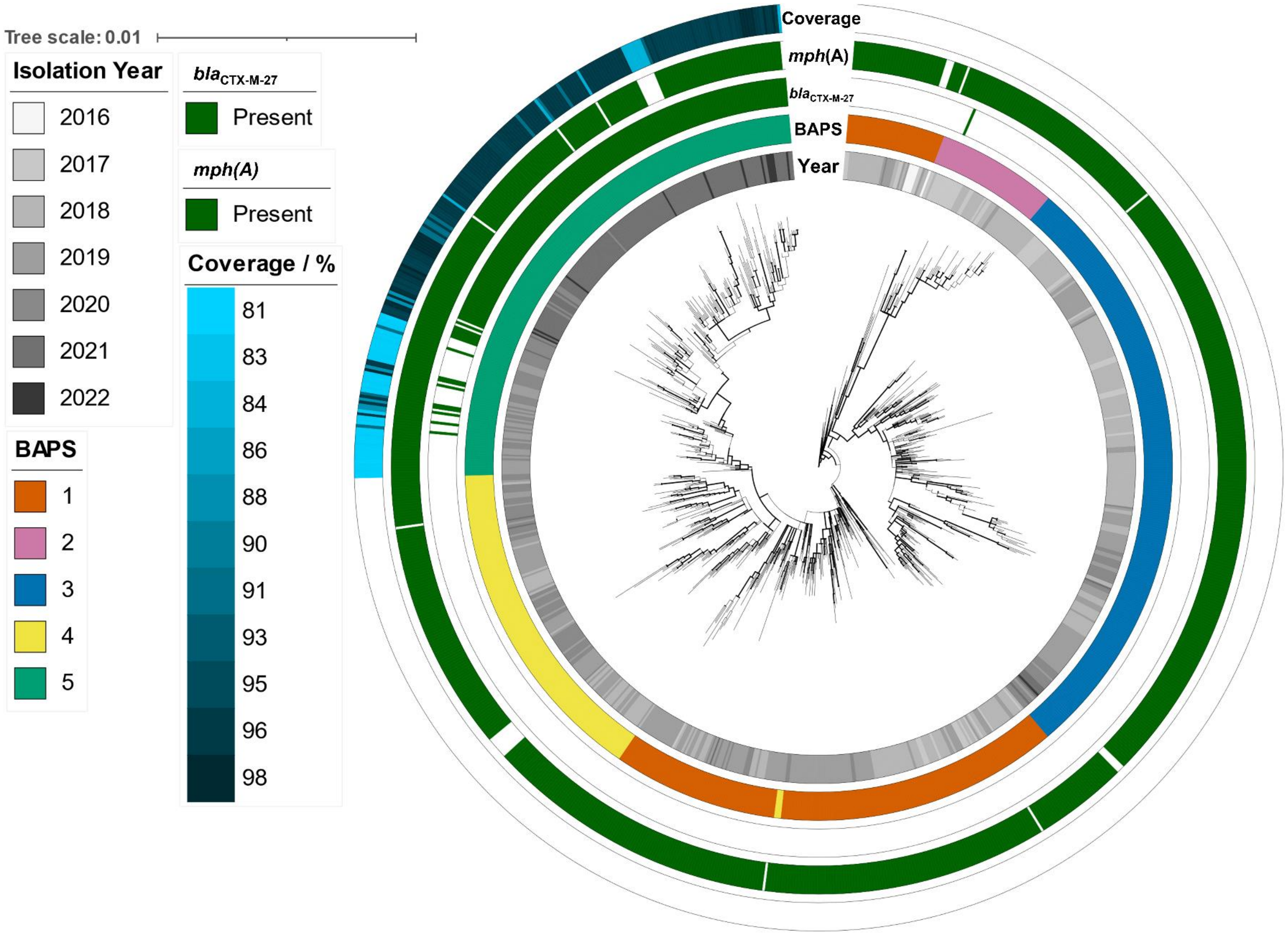

Supplementary Figure 5. p893816 plasmid coverage analyses. A midpoint rooted maximum likelihood phylogenetic tree shows the distribution of the t10.377/CipR.MSM5 subtype of UK *S. sonnei* and selected international isolates. Metadata tracks show year, BAPS group, presence of the $bla_{\text{CTX-M-27}}$ and *mph*(A) genes, and percentage coverage when mapped against p893816 (BAPS 5 isolates only) calculated using Qualimap.[23] The alignment was 1,717 bp. The scalebar is provided by IQTree,[15] and represents the expected number of substitutions per site.

Supplementary Reference List

1 European Committee on Antimicrobial Susceptibility Testing (EUCAST). *Clinical breakpoints and dosing of antibiotics - bacteria (v 12.0)*, <https://www.eucast.org/clinical_breakpoints/> (2022).

2 Malaka De Silva, P. *et al.* A tale of two plasmids: contributions of plasmid associated phenotypes to epidemiological success among *Shigella*. *Proceedings of the Royal Society B: Biological Sciences* **289**, 20220581, doi:10.1098/rspb.2022.0581 (2022).

3 Baker, K. S. *et al.* Horizontal antimicrobial resistance transfer drives epidemics of multiple *Shigella* species. *Nature Communications* **9**, 1462, doi:10.1038/s41467-018-03949-8 (2018).

4 Danecek, P. *et al.* Twelve years of SAMtools and BCFtools. *Gigascience* **10**, doi:10.1093/gigascience/giab008 (2021).

5 Alikhan, N.-F., Petty, N. K., Ben Zakour, N. L. & Beatson, S. A. BLAST Ring Image Generator (BRIG): simple prokaryote genome comparisons. *BMC Genomics* **12**, 402, doi:10.1186/1471-2164-12-402 (2011).

6 Li, H. & Durbin, R. Fast and accurate short read alignment with Burrows-Wheeler transform. *Bioinformatics* **25**, 1754-1760, doi:10.1093/bioinformatics/btp324 (2009).

7 Gilchrist, C. L. M. & Chooi, Y. H. Clinker & clustermap.js: Automatic generation of gene cluster comparison figures. *Bioinformatics*, doi:10.1093/bioinformatics/btab007 (2021).

8 Zhou, Z. *et al.* GrapeTree: visualization of core genomic relationships among 100,000 bacterial pathogens. *Genome Research* **28**, 1395-1404, doi:10.1101/gr.232397.117 (2018).

9 Zhou, Z., Alikhan, N. F., Mohamed, K., Fan, Y. & Achtman, M. The EnteroBase user's guide, with case studies on *Salmonella* transmissions, *Yersinia pestis* phylogeny, and *Escherichia* core genomic diversity. *Genome Research* **30**, 138-152, doi:10.1101/gr.251678.119 (2020).

10 Hunt, M. *et al.* Circlator: automated circularization of genome assemblies using long sequencing reads. *Genome Biology* **16**, 294, doi:10.1186/s13059-015-0849-0 (2015).

11 GNU. *Bash* <https://www.gnu.org/software/bash/> (2007).

12 Croucher, N. J. *et al.* Rapid phylogenetic analysis of large samples of recombinant bacterial whole genome sequences using Gubbins. *Nucleic Acids Research* **43**, e15, doi:10.1093/nar/gku1196 (2015).

13 Nanopore Technologies. *Guppy* <https://nanoporetech.com/> (2022).

14 Letunic, I. & Bork, P. Interactive Tree Of Life (iTOL) v5: an online tool for phylogenetic tree display and annotation. *Nucleic Acids Research* **49**, W293-W296, doi:10.1093/nar/gkab301 (2021).

15 Nguyen, L. T., Schmidt, H. A., von Haeseler, A. & Minh, B. Q. IQ-TREE: a fast and effective stochastic algorithm for estimating maximum-likelihood phylogenies. *Molecular Biology and Evolution* **32**, 268-274, doi:10.1093/molbev/msu300 (2015).

16 Li, H. Minimap2: pairwise alignment for nucleotide sequences. *Bioinformatics* **34**, 3094-3100, doi:10.1093/bioinformatics/bty191 (2018).

17 Ewels, P., Magnusson, M., Lundin, S. & Käller, M. MultiQC: summarize analysis results for multiple tools and samples in a single report. *Bioinformatics* **32**, 3047-3048, doi:10.1093/bioinformatics/btw354 (2016).

18 Hunt, M. *et al.* Antibiotic resistance prediction for *Mycobacterium tuberculosis* from genome sequence data with Mykrobe. *Wellcome Open Research* **4**, 191, doi:10.12688/wellcomeopenres.15603.1 (2019).

19 Feldgarden, M. *et al.* AMRFinderPlus and the Reference Gene Catalog facilitate examination of the genomic links among antimicrobial resistance, stress response, and virulence. *Scientific Reports* **11**, 12728, doi:10.1038/s41598-021-91456-0 (2021).

20 Broad Institute. *Picard Toolkit v. 2.27.2* <https://broadinstitute.github.io/picard/> (GitHub Repository, 2019).

21 Walker, B. J. *et al.* Pilon: An Integrated Tool for Comprehensive Microbial Variant Detection and Genome Assembly Improvement. *PLOS ONE* **9**, e112963, doi:10.1371/journal.pone.0112963 (2014).

22 Seemann, T. Prokka: rapid prokaryotic genome annotation. *Bioinformatics* **30**, 2068-2069, doi:10.1093/bioinformatics/btu153 (2014).

23 García-Alcalde, F. *et al.* Qualimap: evaluating next-generation sequencing alignment data. *Bioinformatics* **28**, 2678-2679, doi:10.1093/bioinformatics/bts503 (2012).

24 Gurevich, A., Saveliev, V., Vyahhi, N. & Tesler, G. QUAST: quality assessment tool for genome assemblies. *Bioinformatics* **29**, 1072-1075, doi:10.1093/bioinformatics/btt086 (2013).

25 RCoreTeam. *R: A language and environment for statistical computing*, <https://www.R-project.org/> (2022).

26 Tonkin-Hill, G., Lees, J., Bentley, S., Frost, S. & Corander, J. RhierBAPS: An R implementation of the population clustering algorithm hierBAPS. *Wellcome Open Research* **3**, 93, doi:10.12688/wellcomeopenres.14694.1 (2018).

27 Free Software Foundation. *GNU sed* <https://www.gnu.org/software/sed/> (2013).

28 SRA Toolkit Development Team. *SRA Toolkit <https://trace.ncbi.nlm.nih.gov/Traces/sra/sra.cgi?view=software>*, 2022).

29 Holt, K., Hawkey, J., Paranagama, K. *Sonneityping script parses Mykrobe predict results for Shigella sonnei* <https://github.com/katholt/sonneityping> (2021).

30 Rambaut, A., Lam, T. T., Max Carvalho, L. & Pybus, O. G. Exploring the temporal structure of heterochronous sequences using TempEst (formerly Path-O-Gen). *Virus Evolution* **2**, doi:10.1093/ve/vew007 (2016).

31 Bolger, A. M., Lohse, M. & Usadel, B. Trimmomatic: a flexible trimmer for Illumina sequence data. *Bioinformatics* **30**, 2114-2120, doi:10.1093/bioinformatics/btu170 (2014).

32 Wick, R. R., Judd, L. M., Gorrie, C. L. & Holt, K. E. Unicycler: Resolving bacterial genome assemblies from short and long sequencing reads. *PLOS Computational Biology* **13**, e1005595, doi:10.1371/journal.pcbi.1005595 (2017).
