# Supplementary material for: The evolution and international spread of extensively drug resistant Shigella sonnei
Source: Nat Commun. 2023 Apr 8;14:1983. doi: 10.1038/s41467-023-37672-w (PMC10082799; doi:10.1038/s41467-023-37672-w)
Supplement: Supplementary file 3 — Description of Additional Supplementary Files [file 41467_2023_37672_MOESM3_ESM.pdf]

Description of Additional Supplementary Files

File Name: Supplementary Data 1
Description: Accession numbers, epidemiological metadata, genotypic and phylogenetic subtyping, and gene presence for all isolates in this study.
