# Supplementary material for: The evolution and international spread of extensively drug resistant Shigella sonnei
Source: Nat Commun. 2023 Apr 8;14:1983. doi: 10.1038/s41467-023-37672-w (PMC10082799; doi:10.1038/s41467-023-37672-w)
Supplement: Supplementary file 5 — Reporting Summary [file 41467_2023_37672_MOESM5_ESM.pdf]

nature portfolio

Corresponding author(s): Professor Kate S Baker

Last updated by author(s): 2023/02/01

Reporting Summary

Nature Portfolio wishes to improve the reproducibility of the work that we publish. This form provides structure for consistency and transparency in reporting. For further information on Nature Portfolio policies, see our Editorial Policies and the Editorial Policy Checklist.

Statistics

For all statistical analyses, confirm that the following items are present in the figure legend, table legend, main text, or Methods section.

n/a	Confirmed	
☐	☒	The exact sample size (*n*) for each experimental group/condition, given as a discrete number and unit of measurement
☐	☒	A statement on whether measurements were taken from distinct samples or whether the same sample was measured repeatedly
☐	☒	The statistical test(s) used AND whether they are one- or two-sided<br>*Only common tests should be described solely by name; describe more complex techniques in the Methods section.*
☒	☐	A description of all covariates tested
☒	☐	A description of any assumptions or corrections, such as tests of normality and adjustment for multiple comparisons
☐	☒	A full description of the statistical parameters including central tendency (e.g. means) or other basic estimates (e.g. regression coefficient) AND variation (e.g. standard deviation) or associated estimates of uncertainty (e.g. confidence intervals)
☐	☒	For null hypothesis testing, the test statistic (e.g. *F*, *t*, *r*) with confidence intervals, effect sizes, degrees of freedom and *P* value noted<br>*Give P values as exact values whenever suitable.*
☒	☐	For Bayesian analysis, information on the choice of priors and Markov chain Monte Carlo settings
☒	☐	For hierarchical and complex designs, identification of the appropriate level for tests and full reporting of outcomes
☒	☐	Estimates of effect sizes (e.g. Cohen's *d*, Pearson's *r*), indicating how they were calculated

Our web collection on statistics for biologists contains articles on many of the points above.

Software and code

Policy information about availability of computer code

Data collection	Illumina paired end reads for the isolates from the United Kingdom were downloaded from the Sequence Read Archive (SRA) using fastq-dump (SRA toolkit v 2.11.0) with default settings except the '—split 3' parameter was used to automatically separate paired end read data into two separate FASTQ files.

Data analysis

A dendrogram was constructed using the NINJA NJ tree algorithm (v 1.0) on the cgMLST hierarchical clustering results from the algorithm implemented in Enterobase (v 1.1.3). Nucleotide sequences were trimmed using Trimmomatic (v 0.39). Quality of trimmed reads was investigated with MULTIQC (v 1.12). Burrows-Wheeler Aligner (BWA v 0.7.17) was used to align the *S. sonnei* sequences to *Shigella sonnei* 53G as the reference genome (NCBI accession number: GCA_00283715.l_ASM28371vl). Draft genomes were assembled using Unicycler (v 0.5.0 and v 0.4.8, as indicated in the manuscript). Draft genome assemblies were checked for quality using QUAST (v 5.02). Draft genomes were analysed for AMR genes using NCBI-AMRFinderPlus (v 3.10.24). SonneiTyping Script (v 20210201) embedded in Mykrobe (v 0.11.0) was used for genotyping and retrieval of QRDR mutations. PICARD (v 2.27.2) was used to mark and remove artificial nucleotide duplicates from the genomes of the *S. sonnei* isolates. SAMTOOLS (v 1.11) was used to index the files. BCFTOOLS (v 1.9) was used to call and filter variants. A chromosomal pseudogenome was created for all isolates and prophages and plasmids were masked using sed (v 4.2.2). Gubbins (v 3.2.1) was then used to remove remaining regions of recombination. Multiple sequence alignments were then used to impute a phylogenetic tree using IQTREE (v 2.2.0.3) and determine population clusters using Rhier Bayesian Analysis of Genetic Population Structure (RhierBAPS v 1.01). Plasmids were re-orientated to start with the repA gene using circulator Fixstart (v 1.5.5), followed by annotation using Prokka (v 1.14.6). A BRIG (v0.95) plot was constructed using default settings with p893816 (GenBank accession MW396858) as the comparator plasmid. R (v 4.1.2) package Growthcurver was used in the plasmid fitness cost analyses. Base-calling was performed post sequencing using Guppy (v 3.4.S+flfbfb) with the New South Wales Oxford Nanopore isolates, with hybrid assembly being performed using Unicycler (v 0.4.8). Assemblies were further polished on Pilon (v 1.23). BAM files generated by MiniMap2 (v 2.17-r941). Assemblies were automatically annotated using Prokka (v 1.14.6). Phylogenetic trees were visualised in the Interactive Tree of Life (iTOL, v 5) website. Clinker (v 0.0.21) was used to compare and visualise plasmid nucleotide identicality. TempEst (v 1.5.3) was used for temporal signal analyses. GNU bash (4.3.30) was used for the command line. Please see the methods section for further detail. Mapping quality for plasmid similarity purposes was analysed using Qualimap (v 2.2.2).

For manuscripts utilizing custom algorithms or software that are central to the research but not yet described in published literature, software must be made available to editors and reviewers. We strongly encourage code deposition in a community repository (e.g. GitHub). See the Nature Portfolio guidelines for submitting code & software for further information.

Data

Policy information about availability of data

All manuscripts must include a data availability statement. This statement should provide the following information, where applicable:

- Accession codes, unique identifiers, or web links for publicly available datasets
- A description of any restrictions on data availability
- For clinical datasets or third party data, please ensure that the statement adheres to our policy

Sequence data relating to all isolates has been deposited in the Sequence Read Archive under BioProject numbers PRJNA315192 (United Kingdom), PRJEB44801 (France), PRJEB40097 (Belgium), and PRJNA613115 (Australia, New South Wales). Individual isolate accession numbers and isolate metadata are available in the Supplementary Data as indicated in the text. Accessions for p893816-like plasmids used in this study are as follows: MW396858.1, CP104412, OP038290, CP115395, LR861790.

Human research participants

Policy information about studies involving human research participants and Sex and Gender in Research.

Reporting on sex and gender

Metadata associated with the *S. sonnei* isolates used in this investigation included 'Sex', which was determined by recorded information on the laboratory submission form for each sample.

Population characteristics

Human research participants were NOT used in this study.

Recruitment

No participants were recruited for this study.

Ethics oversight

Ethical approval was not required for this study as no patient interaction or participation took place. This work was conducted as part of ongoing public health investigations in a partnership with UKHSA.

Note that full information on the approval of the study protocol must also be provided in the manuscript.

Field-specific reporting

Please select the one below that is the best fit for your research. If you are not sure, read the appropriate sections before making your selection.

☒ Life sciences ☐ Behavioural & social sciences ☐ Ecological, evolutionary & environmental sciences

For a reference copy of the document with all sections, see nature.com/documents/nr-reporting-summary-flat.pdf

Life sciences study design

All studies must disclose on these points even when the disclosure is negative.

Sample size

Sample size was determined by data availability, relevance and quality. All samples from the UK with an assigned SNP address (n=2895/2982, 97%) were used in Figure 1 if available on Enterobase (n=2820/2895, 97%). This means that 94.5% (n=2820/2982) of all *S. sonnei* isolates collected by UKHSA between 2016 - 2021 are included in this study, which is a sufficient sample size. Isolates from countries other than the UK were selected based on data availability, relevant, quality and data sharing terms. Please see the methodology for further details regarding criteria of selection for isolates.

Data exclusions

Shigella sonnei isolates from the United Kingdom which had not been assigned a SNP address were excluded. *Shigella sonnei* isolates which were not available on Enterobase were excluded from Figure 1. International isolates which were confirmed by SonneiTyping script to not be CipR.MSM5 were excluded. Please see the methodology for further details regarding criteria of selection for isolates.

Replication	The plasmid fitness cost investigation consisted of three technical replicates and three biological replicates. All attempts at replication were successful.
Randomization	This is not relevant to our study as no human participants were involved in this study, nor was it a clinical trial.
Blinding	This is not relevant to our study as no human participants were involved in this study, nor was it a clinical trial.

Reporting for specific materials, systems and methods

We require information from authors about some types of materials, experimental systems and methods used in many studies. Here, indicate whether each material, system or method listed is relevant to your study. If you are not sure if a list item applies to your research, read the appropriate section before selecting a response.

Materials & experimental systems

n/a	Involved in the study
☒	☐ Antibodies
☒	☐ Eukaryotic cell lines
☒	☐ Palaeontology and archaeology
☒	☐ Animals and other organisms
☒	☐ Clinical data
☒	☐ Dual use research of concern

Methods

n/a	Involved in the study
☒	☐ ChIP-seq
☒	☐ Flow cytometry
☒	☐ MRI-based neuroimaging
